# Therapist memory for treatment contents: Implications for patient outcomes

**DOI:** 10.1016/j.brat.2026.105037

**Published:** 2026-04-06

**Authors:** Catherine A. Callaway, Anne E. Milner, Garret G. Zieve, Leighann Ashlock, Allison G. Harvey

**Affiliations:** Department of Psychology, University of California, 2121 Berkeley Way #1650, Berkeley, CA, 94720-1650, United States.

**Keywords:** Therapist recall, Patient outcomes, Memory support, Cognitive therapy, Depression

## Abstract

**Objective:**

This secondary analysis evaluates the impact of therapist memory on patient outcomes in the context of a randomized trial comparing cognitive therapy plus memory support (CT + Memory Support) to cognitive therapy as usual (CT-as-usual).

**Method:**

Participants were 172 adults (mean age [SD] = 37.46 [15.31]; 62.79% female; 59.88% Caucasian) with major depressive disorder. Patients were treated by 19 therapists (age mean [SD] = 28.4 [5.95] years; 78.95% female; 42.11% Caucasian), with each therapist treating an average of 9 patients. Therapists completed cumulative and past session recall assessments immediately following sessions during weeks 4, 8, and 12, and the final session. Patient depression symptom severity (Inventory for Depressive Symptomatology Self-Report) and functional impairment (World Health Organization Disability Assessment Schedule 2.0) were self-reported at pre-treatment, post-treatment, 6-month (6FU), and 12-month follow-ups (12FU).

**Results:**

Multilevel modeling revealed that better therapist cumulative recall predicted lower depression severity from pre-to post-treatment (unstandardized *b* = −3.43, *p* < 0.001) and at 6FU (*b* = −2.72, *p* < 0.001). Therapist cumulative recall predicted improved patient functional impairment in CT-as-usual only (*b* = 3.11, *p* = 0.02). Therapist past session recall (*b* = −2.68, *p* = 0.01) predicted improved functional impairment across treatment conditions. All other comparisons were non-significant, but the majority were in the hypothesized direction.

**Conclusions:**

These results signal a relationship between therapist memory and patient outcomes. Future research is needed to verify these findings and investigate ways to optimize therapist memory for treatment.

## 1. Introduction

Therapist memory for the delivery of treatment content is unlikely to be optimal, particularly in the delivery of evidence-based psychological treatments (EBPTs). EBPT therapy sessions are typically long and cover complex information, which requires therapists to keep track of different sources of information (e.g., symptoms, case formulation, and content discussed in previous sessions). Therapists also carry a large caseload (Bailey et al., 2021), which means therapists must remember this information across many different clients. More generally, human memory has a limited capacity and is fallible (Camos, 2015; Loftus & Loftus, 2019). Together, these factors make it plausible that therapists may not consistently retain and recall key treatment information from one session to the next.

The limitations of therapist memory may have potential implications for patient outcomes. We offer three key elements of the therapy process that may be implicated, all of which must be subject to further empirical investigation. First, the therapeutic alliance depends in part on consensus between the therapist and patient on the goals and tasks of therapy throughout the therapy course (Vaz et al., 2024), which may benefit from better therapist memory of treatment sessions. For example, one study found that when therapist recall of session contents was more accurate, participants’ perceptions of the therapist were significantly more favorable (Gardner et al., 1988). Second, an individualized case conceptualization requires careful encoding and retention of the patient’s idiosyncratic data of the course of treatment on the part of the therapist, as it is (a) iteratively adapted as sessions unfold and new information becomes available and (b) individualized to each patient’s history and presenting problem (Galovski et al., 2024). Given that case conceptualization guides intervention selection and treatment planning for many EBPTs (Persons & Talbot, 2019), strong therapist recall may be crucial for diagnostic judgments, development and testing of case formulations, treatment planning, and selection of therapeutic strategies (Brailey et al., 2001; Strohmer et al., 1990; Webb et al., 2016). Lastly, repetition of key treatment points is key to lasting change in patient behavior. Thus, if a therapist cannot recall key treatment points and provide the repetition necessary to promote habit formation in their patients, the potential for sustained treatment outcomes may be limited (Harvey et al., 2022). These three key elements may be strengthened when therapists retain and recall treatment points and session information; therapy improves treatment delivery and subsequently patient outcomes.

Importantly, therapist memory for treatment is modifiable. There is research documenting that memory encoding and retention can be markedly improved via the application of memory support strategies. Harvey et al. (2014) sought to harness these strategies to engage *patient* memory for EBPTs via the Memory Support Intervention. The goal of the Memory Support Intervention is to increase patient memory for key treatment points, with the goal of improving treatment adherence and outcomes. A treatment point is defined as an insight, skill, or strategy that the therapist thinks is important for the patient to remember and/or implement as part of the treatment (Lee & Harvey, 2015). Although the Memory Support Intervention is delivered to enhance patient memory, it may also strengthen therapist memory. Specifically, delivering memory support strategies requires the therapist to also highlight and repeat treatment content to the patient, which is likely to strengthen the therapist’s own encoding and later retrieval of the same material. Indeed, evidence suggests that the Memory Support Intervention also improves *therapist* memory for key treatment points, such that therapist memory improved in the CT + Memory Support condition, relative to the CT-as-usual (Callaway et al., submitted).

In the current study, we focus on two distinct measures of therapist recall, namely, past-session and cumulative recall for treatment points, which are defined as “insights, skills, or strategies that you [the therapist] think are important for your patient to remember and/or implement as a part of treatment.” Past-session recall refers to the therapist’s memory of treatment points from the immediately preceding session, whereas cumulative recall reflects memory for treatment points across the full course of therapy to date (cumulative recall). These forms of recall may influence outcomes through different mechanisms. Prior-session recall could support the reinforcement of key treatment points, and alignment and goals from session-to-session. In contrast, cumulative recall may be more closely aligned with individualized case formulation and integration of skills over time, which may strengthen the therapeutic alliance over time. Therefore, cumulative recall may be particularly relevant for tailoring and improving treatment, which may further sustain patient outcomes.

The present study was designed to evaluate whether therapist recall predicts patient outcomes in the context of a randomized trial comparing cognitive therapy plus memory support (CT + Memory Support) to cognitive therapy as usual (CT-as-usual) in adults diagnosed with major depressive disorder (MDD). Specifically, we examined whether therapist recall predicted changes in patient outcomes (depression symptom severity and functional impairment) from pre-treatment (PRE) to post-treatment (POST), and whether any improvements are sustained to follow-up time points at 6-month follow-up (6FU), and 12-month follow-up (12FU). We also examined whether these effects are moderated by treatment condition (CT-as-usual vs. CT + Memory Support). We hypothesize that better therapist recall will be associated with improved patient outcomes. Furthermore, we expect this relationship to be stronger in the CT + Memory Support condition relative to CT-as-usual. As the Memory Support Intervention is expected to enhance encoding of key treatment points, it may strengthen therapist recall in ways that directly support the therapeutic alliance and individualized case conceptualization. As a result, variability in therapist recall in the CT + Memory Support condition may translate more strongly to patient improvement.

## 2. Method

### 2.1. Study overview and participants

Data for the current study were drawn from a randomized controlled trial (NCT02938559) funded by the National Institute for Mental Health (R01MH108657; Dong et al., 2022). Adults who met criteria for major depressive disorder (*N* = 178) were randomly assigned in a 1:1 parallel group design to CT + Memory Support (*n* = 87) or CT-as-usual (*n* = 91). Randomization was stratified by age (≤49, 50+) and depression chronicity (<2 yrs, ≥2 yrs; Fournier et al., 2009). Additional details about the eligibility and procedures for the trial are described elsewhere (Dong et al., 2022). Six participants were excluded from the present study because they were missing at least one of the key measures at all time-points, leaving 172 participants treated by 19 therapists (88 CT + Memory Support, 84 CT-as-usual) included in these analyses. The 19 therapists treated an average of 9.05 patients (*SD* = 6.70, range = 1 to 25). All participants provided informed consent to participate. This study received approval from the University of California, Berkeley, Committee for the Protection of Human Subjects.

### 2.2. Treatments

Treatment was administered by a licensed therapist or graduate students in social work or clinical psychology. Both CT + Memory Support and CT-as-usual were comprised of 20 to 26, 50-min sessions conducted over 16 weeks. Patients received twice-weekly sessions for weeks 1-4, one to two sessions per week for weeks 5-10, and once-weekly sessions for weeks 11-16. All sessions were video recorded. To help ensure purity of delivery (Manber et al., 2008), each therapist was randomly allocated to deliver only one of the two treatment conditions, and only those therapists in the CT + Memory Support condition were trained to deliver memory support strategies (see below). For both treatment conditions, therapists delivered CT according to an identical protocol and received weekly supervision to standardize treatment administration. Weekly supervision was conducted separately for therapists in each condition, except that all therapists attended monthly master classes with a licensed therapist.

#### 2.2.1. CT-as-usual

CT was developed by Beck (1979) and has incorporated several innovations (Beck, 1995; Bennett-Levy et al., 2004). Treatment strategies are designed to identify, reality test, and correct distorted beliefs and information processing (Beck, 1979). CT for major depressive disorder was conducted according to the standard manuals (Beck, 1979, 2011; Greenberger & Padesky, 2015). Patients were also given a copy of a CT self-help book (Greenberger & Padesky, 2015).

#### 2.2.2. CT + memory support

The Memory Support Intervention is a manualized adjunctive treatment designed to be delivered alongside CT-as-usual. Therapists in the CT + Memory Support condition were trained to deliver strategies from the Memory Support Intervention and were instructed to incorporate these strategies as much as possible during sessions. The Memory Support Intervention is comprised of eight memory promoting strategies, with four constructive and four non-constructive strategies (see Table 1). These strategies are proactively, strategically, and intensively integrated into treatment-as-usual to support memory encoding. Memory support was delivered alongside each ‘treatment point’, defined as a main idea, principle, or experience that the therapist wants the patient to remember or implement as part of the treatment (Lee & Harvey, 2015). We acknowledge that several recommended practices within CT-as-usual may function as memory support strategies (e.g., providing regular capsule summaries may function as repetition). Thus, there was also some level of memory support delivered in the CT-as-usual condition. However, as described previously (Dong et al., 2022), therapists in CT + Memory Support delivered significantly more memory support, as well as a greater number of different types of memory support strategies, compared to therapists in CT-as-usual.

### 2.3. Measures

In addition to therapist and patient demographics, the following measures were collected.

#### 2.3.1. Therapist measures

##### Therapist Recall Task.

Therapist memory for treatment was measured using the Therapist Recall Task (TRT), a variation of the Patient Recall Task (PRT; Lee & Harvey, 2015). The TRT is a free recall measure of therapist memory, during which therapists are given a sheet of paper and asked to take 10 min to recall as many distinct treatment points as they can from the immediately preceding session and all the sessions they have had thus far up until the time of the assessment (“cumulative recall”) as well as from the immediately preceding session only (“past session recall”). Treatment points are defined for therapists as “insights, skills, or strategies that you [the therapist] think are important for your patient to remember and/or implement as a part of treatment.” Examples of therapist treatment points include “thoughts, behaviors, and physiology affect our mood” and “thinking traps include black and white thinking.” The overall number of treatment points recalled was coded by a trained coding team using a scoring rubric developed in a previous study (Lee & Harvey, 2015). According to the rubric, recall responses must be consistent with CT to count towards the overall total. The raw number of treatment points recalled by the therapist are then summed. If a therapist writes the same idea more than once, one point is awarded to the group of responses. To assess inter-rater reliability, each member of the coding team individually coded the number of treatment points from 10% of TRT tasks collected (reflecting a total of 69 TRT tasks). Interrater reliability was excellent according to a two-way mixed effects, consistency, single rater intraclass correlation (n = 69, ICC = 0.93, *p* < 0.001) (McGraw & Wong, 1996). Therapists completed the TRT immediately following sessions that occurred during weeks 4, 8, and 12 and the final session, to capture therapist memory throughout treatment and following all treatment sessions.

#### 2.3.2. Patient outcome measures

To limit the number of comparisons, one patient outcome measure was selected from the parent study (NCT02938559) for each domain (course of illness, functional impairment) to include in the present analyses. A rationale for measure selection is provided below.

##### Course of illness.

The Inventory for Depressive Symptomatology Self-Report (IDS-SR) (Trivedi et al., 2004) was used to measure the course of illness. The IDS-SR is a 30-item scale used to measure depression severity. Each item is rated on a 4-point scale ranging from 0 (symptom is not present) to 3 (strongest impairment). Items were summed with higher scores indicating more severe symptoms. The IDS-SR has demonstrated high internal consistency (Cronbach’s α = 0.92 to 0.93) (Trivedi et al., 2004), and internal consistency estimates for the current sample were high across all timepoints (Cronbach’s α = 0.75 to 0.93). We selected the continuous score of the IDS-SR for the present analyses, as opposed to binary outcome measures (e.g., remission, relapse), as it provides a wider range of possible courses of illness in response to treatment.

##### Functional Impairment.

The World Health Organization Disability Assessment Schedule 2.0 (WHODAS 2.0) (Üstün et al., 2010) is a 36-item scale used to assess difficulty in specific areas of functioning from the past 30 days. Items are scored on a 5-point scale from 1 (‘none’) to 5 (‘extreme or cannot do’). Summary scores of the WHODAS were derived based on simple scoring instruction (Üstün et al., 2010). Higher scores indicate greater disability. The WHODAS has demonstrated high internal consistency (Cronbach’s α = 0.86) and test-retest reliability (Üstün et al., 2010). Internal consistency estimates for the current sample were high across all timepoints (Cronbach’s α = 0.92 to 0.94). We selected the WHODAS as it was the primary outcome measure for functional impairment used in the parent study.

### 2.4. Data analysis

One to six percent of data was missing across measures for participants included in the following analyses. Maximum likelihood (ML) estimation method was used with missing data assumed Missing at Random (MAR). All data analyses were conducted in R (R Core Team, 2016). For the continuous outcome measures (IDS-SR, WHODAS), multilevel modeling was used to evaluate the effect of therapist cumulative recall and past session recall on outcome measures post-treatment, 6FU, and 12FU (with pre-treatment as reference) by treatment condition (Goldstein, 2011; Hox et al., 2017; Raudenbush & Bryk, 2002). All continuous predictor variables (i.e., IDS-SR, WHODAS, therapist recall) and covariates were z-scored to improve model convergence and improve interpretation of the coefficients. The first level represents within-person (patient-level) variation and includes time indicators (or dummy variables) as predictors (post-treatment, 6FU, 12FU, with pre-treatment as the reference). The second level represents between-person variation in the intercepts and coefficients and includes treatment condition (CT + Memory support, CT-as-usual as reference) and therapist cumulative recall or therapist past-session recall as a continuous predictor variable. Therapist recall was modeled at the patient level. Although therapists contributed multiple observations per patient, these observations were collapsed into a single therapist-recall score per patient. We initially specified a nested random-effects structure with patients nested within therapists. However, these models were singular (therapist intercept variance = 0), indicating negligible between-therapist variance or an insufficient number of therapists to estimate this variance reliably. Therefore, therapist-level random effects were excluded, and the final models only retained the patient random intercept. The following therapist baseline characteristics were included as covariates, as they may account for variations in treatment delivery: age, years of education, prior CBT experience, and number of patients treated. In addition, patient recall was also included as a covariate as patient recall has previously been shown to be improved in the CT + Memory Support condition, relative to CT-as-usual (Dong et al., 2022). Pairwise comparisons using the emmeans package in R were used to follow up on significant interactions. The Benjamini-Hochberg procedure (Benjamini & Hochberg, 1995) was used to correct for multiple comparisons for the follow-up tests using a 5% false discovery rate. An alpha level of 0.05 was used.

### 2.5. Transparency and openness

All research materials, data, and analysis code are available from the authors upon request. This study was not pre-registered. We report all data exclusions, all manipulations, and all measures in the study.

## 3. Results

Demographic variables for therapists are presented in Table 2 and for patients in Table 3. Table 4 displays the descriptive statistics for all measures included in the present analyses at each timepoint. Table 5 reports the multilevel modeling results for therapist cumulative recall and past-session recall on the changes in IDS-SR and WHODAS scores from pre-treatment to post-treatment, 6FU, and 12FU. This table includes both the random effect estimates and the fixed effects for the two primary interactions of interest: (1) the two-way interaction between therapist recall and timepoint indicators, which examines whether the association between therapist recall and patient outcome changes over time, and (2) the three-way interaction between therapist recall, time-point indicators, and treatment condition (CT-as-usual versus CT + Memory Support), which test whether the two-way interaction effect differs by treatment condition. A total of four models are reported below, as the two outcome measures are analyzed with the two distinct types of therapist recall as predictors in separate models.

### 3.1. IDS-SR

Two separate multilevel models were used to assess the effects of therapist cumulative recall and therapist past session recall on the magnitude of symptom change, as indexed by the IDS-SR from pre-treatment to post-treatment, to 6FU, and to 12FU by treatment condition.

#### 3.1.1. Cumulative recall

There was no significant effect of therapist cumulative recall on change in patient IDS-SR scores from pre-treatment to any of the follow-up timepoints (all *p*’s > 0.05). However, there was a significant interaction between therapist cumulative recall and treatment condition on the change in patient IDS-SR scores from pre-treatment to post-treatment (*b* = −3.43, *SE* = 0.82, *p* < 0.001; see Fig. 1a). Pairwise comparisons revealed that better therapist cumulative recall was associated with improved patient depressive symptoms from pre-treatment to post-treatment in the CT + Memory Support condition (*t* = −5.78, *SE* = 15.08, *p* < 0.001) but not the CT-as-usual condition (*t* = 1.16, *SE* = 16.37, *p* = 0.40). There was also a significant interaction between therapist cumulative recall and treatment condition on the change in patient IDS-SR scores from pre-treatment to 6FU (*b* = −2.72, *SE* = 0.82, *p* < 0.001) that followed a similar pattern, such that better therapist cumulative recall was associated with improved patient depressive symptoms from pre-treatment to 6FU in the CT + Memory Support condition (*t* = −4.57, *SE* = 14.81, *p* < 0.001) but not the CT-as-usual condition (*t* = 0.98, *SE* = 16.36, *p* = 0.40). There was no significant interaction between therapist cumulative recall and treatment condition on IDS-SR scores from pre-treatment to 12FU (*b* = −1.02, *SE* = 0.83, *p* = 0.22), suggesting that the benefits of improved cumulative memory recall in the CT + Memory Support condition were no longer observed at 12FU.

#### 3.1.2. Past session recall

There was no significant effect of therapist past-session recall on change in patient IDS-SR scores from pre-treatment to any of the follow-up timepoints (all *p*’s > 0.05). There were also no significant interactions between therapist past-session recall and treatment condition on the change in patient IDS-SR scores from pre-treatment to post-treatment (*b* = −1.38, *SE* = 0.78, *p* = 0.08; see Fig. 1b), to 6FU (*b* = -−0.63, *SE* = 0.78, *p* = 0.42), or to 12FU (*b* = −0.87, *SE* = 0.81, *p* = 0.28), suggesting that improved therapist past-session recall did not affect depressive symptoms.

### 3.2. WHODAS

Two separate multilevel models were used to assess the effects of therapist cumulative recall and therapist past session recall on functional impairment as measured by the WHODAS from pre-treatment to post-treatment, to 6FU, and to 12FU by treatment condition.

#### 3.2.1. Cumulative recall

There was no significant effect of therapist cumulative recall on change in patient IDS-SR scores from pre-treatment to post-treatment (*b* = 0.74, *SE* = 1.09, *p* = 0.49) or to 6FU (*b* = −1.84, *SE* = 1.08, *p* = 0.09). There was a significant effect of therapist cumulative recall on WHODAS scores from pre-treatment to 12FU (*b* = −3.09, *SE* = 1.12, *p* < 0.01; see Fig. 1c); this effect was such that improved therapist cumulative recall was associated with improved WHODAS scores from pre-treatment to 12FU. However, this effect was superseded by a higher order interaction with treatment condition (*b* = 3.11, *SE* = 1.33, *p* = 0.02), that indicated that the effect of therapist cumulative recall was significant in the CT-as-usual condition (*t* = −2.78, *SE* = −26.77, *p* = 0.02) but not in the CT + Memory Support condition (*t* = 0.68, *SE* = 23.83, *p* = 0.98).

#### 3.2.2. Past session recall

There was no significant effect of therapist past-session recall on change in patient WHODAS scores from pre-treatment to post-treatment (*b* = 0.45, *SE* = 1.00, *p* = 0.66), or to 6FU (*b* = −1.57, *SE* = 1.00, *p* = 0.12). However, there was a significant effect of therapist past-session recall on the change in WHODAS scores from pre-treatment to 12FU (*b* = −2.67, *SE* = 1.05, *p* = 0.01; see Fig. 1d), which indicated that improved therapist past-session recall was associated with improved WHODAS scores from pre-treatment to 12FU. There was also no significant interaction between therapist past-session recall and treatment condition on change in patient WHODAS scores from pre-treatment through all follow-up timepoints (all *p*’s > 0.05).

## 4. Discussion

The overarching goal of this paper was to continue progressing knowledge of the relationship between therapist memory and patient outcomes. Specifically, this study sought to evaluate if therapist recall of treatment contents predicts patient outcomes. This is an important line of inquiry because the limitations of therapist memory are significant (e. g. Webb et al., 2016) and may adversely impact treatment outcome (e.g. Brailey et al., 2001; Strohmer et al., 1990). Furthermore, therapist memory for treatment contents is modifiable (Callaway et al., submitted; Harvey et al., 2014) and may offer a possible intervention target to better patient outcomes across various EBPTs (pantreatment) and diagnoses (transdiagnostic).

The context for the present study was a randomized trial in which therapists delivered CT to adults diagnosed with MDD. Our aim was to examine whether therapist recall predicted patient outcomes at post-treatment (POST), 6-month follow-up (6FU), and 12-month follow-up (12FU), controlling for pre-treatment levels of each patient outcome measure (PRE), and whether this relationship was moderated by treatment condition. Patient outcomes were defined as depression symptom severity and functional impairment. We hypothesized that better therapist recall would predict improved patient outcomes, and that these effects would be moderated by treatment condition.

The relationship between therapist recall and patient depression symptom severity was observed primarily in the CT + Memory Support condition. Specifically, better cumulative recall was associated with greater improvements in patient depression symptom severity from pre-treatment to post-treatment and 6FU, but these improvements were not retained at 12FU. However, better therapist past session recall was not associated with improved depression severity from pre-treatment to any follow-up timepoints. In the CT-as-usual condition, better therapist cumulative recall was associated with improvements in patient functional impairment from pre-treatment to 12FU. Across both treatment conditions, better therapist past session recall was associated with improved patient functional impairment from pre-treatment to 12FU. In sum, three of twelve total comparisons examining the cumulative recall by treatment condition by timepoint interaction across both patient outcome measures and both types of recall were statistically significant in the hypothesized direction. Upon inspecting the beta values for nonsignificant findings, six of the nine remaining comparisons were in the hypothesized direction. Taken together, these results offer a promising preliminary signal that better therapist memory holds potential to improve patient outcomes. Interestingly, most significant findings indicated that therapist memory had a greater effect on patient outcomes in the CT + Memory Support condition relative to the CT-as-usual. This aligns with preliminary evidence showing that the Memory Support Intervention improves therapist memory for key treatment points, which subsequently improves patient outcomes.

The relationship between therapist memory and patient outcomes could be due to many factors. We offer three key elements of the therapy process that may be implicated, all of which must be subject to empirical investigation. First, the therapeutic alliance depends in part on consensus between the therapist and patient on the goals and tasks of therapy throughout the therapy course (Vaz et al., 2024), which may benefit from better therapist memory of treatment sessions. Second, an individualized case conceptualization requires careful encoding and retention of the patient’s idiosyncratic data of the course of treatment on the part of the therapist, as it is (a) iteratively adapted as sessions unfold and new information becomes available and (b) individualized to each patient’s history and presenting problem (Galovski et al., 2024). Given that case conceptualization guides intervention selection and treatment planning for many EBPTs (Persons & Talbot, 2019), strong therapist recall may be crucial for selecting an appropriate course of therapy and making necessary adjustments throughout. Lastly, there is a growing focus on applying the science of habit formation to EBPTs to improve and sustain treatment outcomes (Harvey et al., 2022). A core element of forming a new habit or disrupting an unwanted habit (typically behaviors or thought patterns in a therapy context) is repetition. For the therapist to sufficiently repeat the key treatment points that are central to each patient’s goals, the therapist must first recall these points across sessions.

Interestingly, results across both patient outcome measures appear to be stronger for the impact of therapist cumulative recall compared to therapist past session recall. It is possible that therapist cumulative recall, meaning recall across the entire course of treatment for a given patient, is more central for optimizing patient outcomes compared to therapist recall of each individual session. In other words, the therapist’s ability to remember specific details of a single session may be less important than their ability to remember and integrate patient information over multiple sessions. The key responsibilities of a therapist (planning treatment, selecting therapeutic strategies, developing and testing case formulations, building a strong rapport with the patient) require recognizing patterns that may not be obvious from individual sessions alone. Another explanation for this pattern of findings is that there may have been a ceiling effect for therapist past session recall, reducing variability across therapists and limiting our ability to detect an effect. Indeed, past session recall likely did not tax therapist memory nearly to the same extent as cumulative recall. While cumulative recall required therapists to remember at least two (and up to 24) sessions spanning weeks or months prior, past session recall required therapists to remember only a single session which occurred mere minutes prior. A significant amount of forgetting occurs within an hour of learning new material (Murre & Dros, 2015). Therefore, the timing of the past session recall may have occurred too close to the session being recalled for it to challenge memory enough to reveal significant differences among therapists. Future studies might consider delivering the Therapist Recall Task several hours or a day after the patient session of interest to ensure both past session and cumulative recall are sensitive to variations in therapists’ memory functioning.

This study has several limitations. First, the Therapist Recall Task prompts therapists to recall treatment points that they deem important for the patient to remember (see Measures). Additional research is needed to clarify the content most relevant for therapists to remember to optimize patient outcomes. Second, the accuracy of the points therapists recalled is unknown. Third, the scoring rubric for the Therapist Recall Task (one point for each treatment point recalled), assumes that all treatment points are equally significant. While this counting method in a similar task shows evidence of reliability and validity (Harvey et al., 2016; Lee & Harvey, 2015), there may be important differences in the quality, importance, or impact of recalled treatment points (Dong et al., 2017). Fourth, no cut-off score or benchmark has been identified for the Therapist Recall Task to designate a therapist’s recall of treatment contents as sufficient to support patient outcomes, but rather assumes that the more the therapist remembers, the better. It is possible there is a point of diminishing return regarding the number of treatment points recalled. Fifth, the number of therapist treatment points recalled may serve as a proxy for the number of insights, skills, or strategies implemented by the therapist. However, a key point is that this is likely to be more greatly related to past-session recall, which measures recall for treatment points delivered in the prior session. In contrast, cumulative recall, which had the greatest impact on patient outcomes, would not directly translate to dosage in each session, as it reflects the therapist’s memory for treatment points delivered throughout treatment. Cumulative recall may better reflect individualized case formulation and integration of skills over time, which may strengthen the therapeutic alliance over time and improve treatment delivery. Sixth, this study only examined one type of therapist memory: long-term memory. As new information must pass through short-term memory before being encoded in long-term memory (Baddeley & Hitch, 1974), optimizing the functioning of both types of therapist memory is likely important for patient outcomes. It would be valuable to disentangle the differential effects of these types of therapist memory on patient outcomes in future studies. Finally, although the current analyses ruled out the confounding effects of therapist experience and education, there may be other unmeasured variables related to therapist characteristics that may have influenced patient outcomes, such as therapist engagement and intelligence. Further research is warranted to test these possibilities and to substantiate the relationship between therapist memory and patient outcomes.

In conclusion, these results provide preliminary evidence to support a relationship between therapist memory and patient outcomes. Importantly, this relationship was moderated by treatment condition (CT + Memory Support vs. CT-as-usual), suggesting that this relationship was notably stronger when therapists received memory support. Therapist memory functioning over the course of treatment may have a stronger relationship with patient outcomes than memory functioning immediately following each session. Future research is needed to substantiate these findings and investigate ways to optimize therapist memory for treatment.

## Figures and Tables

**Fig. 1.**
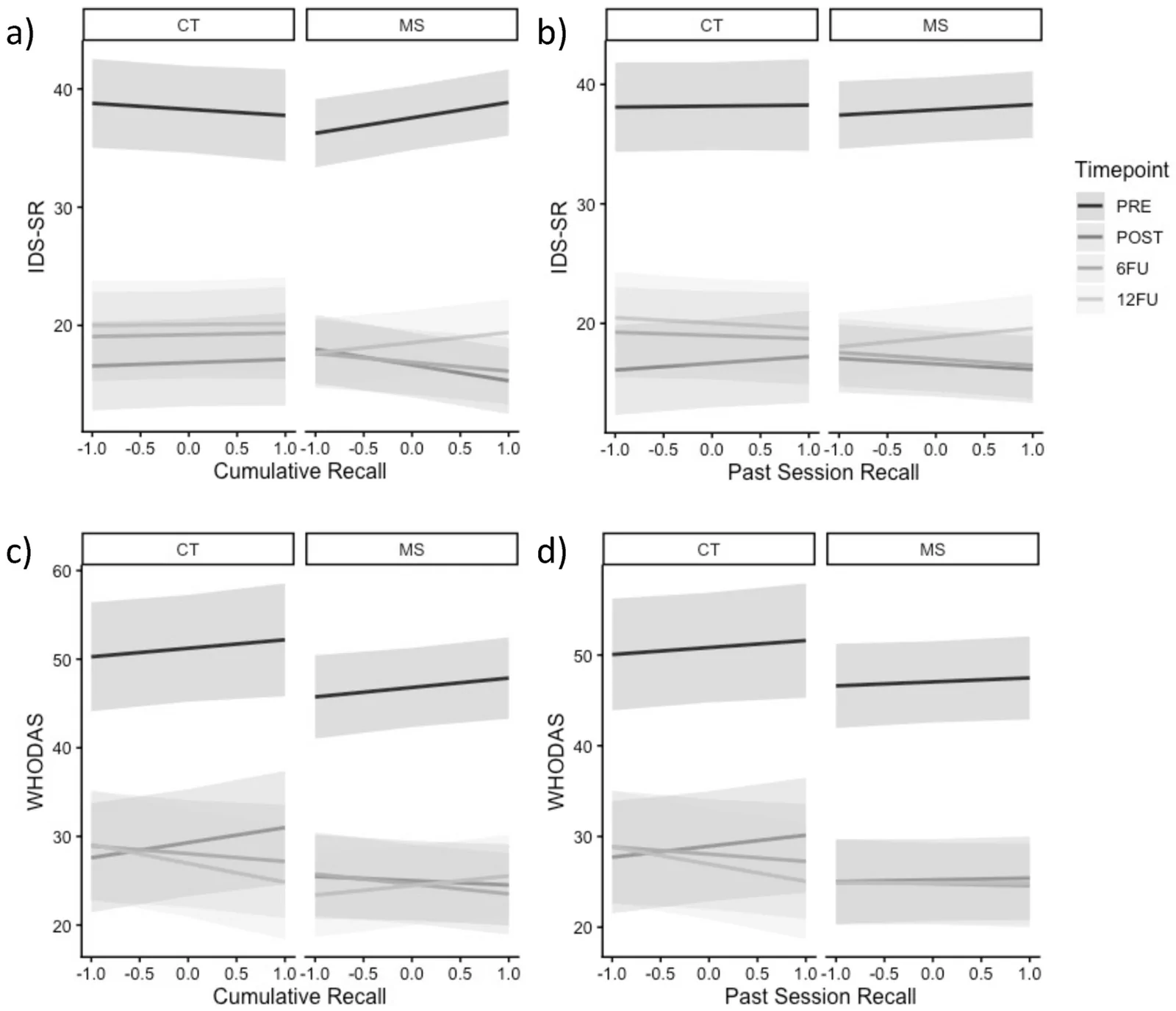
The effect of therapist recall on patient outcomes at pre-treatment, post-treatment, 6FU, and 12FU by treatment condition (CT-as-usual versus CT + Memory Support). a) cumulative recall on IDS-SR, b) past session recall on IDS-SR, c) cumulative recall on WHODAS, d) past session recall on WHODAS. *Note.* Both Cumulative and past session recall were scaled, resulting in a range from −1 to 1. CT = Cognitive Therapy, MS = Memory Support, PRE = pre-treatment, POST = post-treatment, 6FU = 6-month follow-up, 12FU = 12-month follow-up. IDS-SR = Inventory for Depressive Symptomatology Self-Report, WHODAS = World Health Organization Disability Assessment Schedule 2.0.

**Table 1:** List of the eight memory supports.

Strategy	Definition
Attention recruitment	Directing attention to specific therapy points via emphasis or scaffolding
Categorization	Organizing therapy points into distinct categories
Evaluation	Reviewing the pros and cons of using a skill or adopting a belief
Application	Prompting the participant to describe how a treatment may be relevant to their individual life
Repetition	Repeating treatment points for emphasis
Practice remembering	Asking participants questions that prompt recall of treatment points
Cue-based reminder	Preemptively establishing cues to help participant remember to use a skill or consider a treatment point
Praise recall	Reinforcing correct recall of treatment points

**Table 2:** Provider Demographics by Treatment Condition (CT-as-usual vs. CT + Memory Support) at Pre-Treatment.

	CT-as-usual (*n* = 10)		CT + Memory Support (*n* = 9)			
*Therapist Characteristics*	*n*	%	n	%	χ^2^	*p*-value

**Sex**					<0.001	1.00
Female	8	80.00	7	77.80		
Male	2	20.00	2	22.20		
**Ethnicity**					0.82	0.36
Hispanic or Latino	4	40.00	1	1.11		
Not Hispanic or Latino	6	60.00	8	88.90		
**Race**					1.99	0.74
American Indian/Alaska Native	1	10.00	0	0.00		
Asian	2	20.00	1	11.10		
African American or Black	1	10.00	1	11.10		
White	3	30.00	5	55.60		
Multi-Racial	3	30.00	2	22.20		
**Education**					4.56	0.21
Some graduate school	9	90.00	6	66.70		
Competed master’s degree	0	0.00	2	22.22		
Graduate Training beyond master’s	1	10.00	0	0.00		
Completed Doctorate	0	0.00	1	11.10		
**Area of Education/Field**					5.10	0.17
Clinical Psychology	1	80.00	4	44.44		
Social Work	8	10.00	5	55.56		
Education/School Psychology	1	10.00	0	0.00		

	Mean	SD	Mean	SD	t	p-value

**Age (years)**	29.80	7.73	27.00	2.83	1.07	0.31
**Number of patients treated**	8.20	5.43	10.00	8.12	−0.56	0.58
**Prior experience delivery CBT (years)**	0.10	0.32	1.33	2	−1.83	0.10
**Prior experience delivery treatment for depression (years)**	1.25	1.59	1.78	2.17	−0.60	0.56

**Table 3:** Patient Demographics by Treatment Condition (CT-as-usual vs. CT + Memory Support) at Pre-Treatment.

	CT-as-usual (*n* = 88)		CT + Memory Support (*n* = 84)			
*Patient Characteristics*	*n*	%	*n*	*%*	χ^2^	*p*-value

**Sex**					4.20	0.12
Female	49	55.68	59	70.24		
Male	37	42.05	25	29.76		
Missing/declined to answer	2	2.28	0	0.00		
**Ethnicity**					1.00	0.60
Hispanic or Latino	14	15.91	15	17.86		
Not Hispanic or Latino	71	80.68	68	80.95		
Missing/declined to answer	3	3.41	1	1.19	10.33	0.10
**Race**						
American Indian/Alaska Native	0	0.00	1	1.19		
Native Hawaiian/Pacific Islander	1	1.14	0	0.00		
Asian	11	12.50	15	17.86		
African American or Black	1	1.14	5	5.95		
White	51	57.95	52	61.90		
Multi-Racial	23	26.14	10	11.90		
Missing/declined to answer	1	1.14	1	1.19		
**Marital Status**					3.30	0.19
Single	56	63.64	51	60.71		
Married/Partnered	25	28.41	19	22.62		
Divorced/Separated/Widowed	7	7.96	14	16.66		
**Employed**					5.21	0.27
Full-time	34	38.64	29	34.52		
Part-time	26	29.55	16	19.05		
Unemployed	23	26.14	31	36.90		
Retired	5	5.68	7	8.33		
Missing/declined to answer	0	0.00	1	1.19		
**Income (Household)**					3.01	0.70
<$20,000	3	3.41	8	9.52		
$20,000-$34,999	16	18.18	15	17.86		
$35,000-$49,999	9	10.23	7	8.33		
$50,000-$59,999	5	5.68	5	5.95		
≥$60,000	29	32.95	28	3.33		
Missing/declined to answer	26	29.55	21	25.00		
**Education**					0.46	0.93
High school/vocational school	2	2.27	3	3.57		
Some college	27	30.68	27	32.14		
Completed college	26	29.55	22	26.10		
Some graduate school/completed graduate school	33	37.50	32	38.10		

	Mean	SD	Mean	SD	t	p-value

**Age (years)**	37.73	14.45	37.18	16.24	0.23	0.82

**Table 4:** Descriptive statistics for the predictor and patient outcomes.

	CT-as-usual			CT + Memory Support		
Predictor	N	M	SD	N	M	SD

**Therapist Cumulative Recall**						
Mid 1	86	11.24	3.58	82	11.72	4.84
Mid 2	80	12.10	3.02	79	14.38	5.62
Mid 3	76	13.39	4.63	78	14.81	6.29
Post	76	14.07	4.99	80	16.46	7.41
**Therapist Past Session Recall**						
Mid 1	86	6.05	3.31	84	6.04	3.75
Mid 2	83	5.41	3.64	80	6.05	4.30
Mid 3	79	5.89	3.79	81	5.79	4.32
Post	81	6.68	4.30	80	10.53	6.54

Outcome	N	M	SD	N	M	SD

**IDS-SR**						
Pre-treatment	88	39.59	7.61	84	38.40	18.16
Post	81	17.58	12.20	78	16.91	17.07
6FU	81	20.31	12.43	83	17.39	18.83
12FU	75	20.92	12.51	78	19.51	17.90
**WHODAS**						
Pre-treatment	88	48.22	18.16	84	45.55	16.19
Post	81	25.17	17.07	77	23.31	16.26
6FU	80	25.91	18.83	83	23.35	15.55
12FU	75	23.77	17.90	79	23.23	17.77

**Table 5:** Coefficient estimates from multilevel models of IDS-SR and WHODADS for the effect of Therapist Cumulative and Past-Session Recall.

Model 1: Cumulative Recall on IDS-SR				
Random Effects	Variance	SD		
Patient	71.16	8.44		
Residual	43.20	6.57		
Fixed Effects	b	SE	P	95% CI
*Cumulative Recall*				
Pre-treatment to post-treatment	0.79	0.68	0.25	[−0.54, 2.12]
Pre-treatment to 6FU	0.67	0.68	0.33	[−0.66, 2.00]
Pre-treatment to 12 FU	0.58	0.69	0.40	[−0.77, 1.93]
*Cumulative Recall x Treatment Condition*				
Pre-treatment to post-treatment	−3.43	0.82	**< 0.001**	[−5.04, −1.83]
Pre-treatment to 6FU	−2.72	0.82	**< 0.001**	[−4.31, −1.12]
Pre-treatment to 12 FU	−1.02	0.83	0.22	[−2.65, 0.60]

Model 2: Past-Session Recall on IDS-SR				
Random Effects	Variance	SD		
Patient	71.66	8.50		
Residual	43.59	6.60		
Fixed Effects	b	SE	p	95% CI
*Past-Session Recall*				
Pre-treatment to post-treatment	0.47	0.64	0.46	[−0.77, 1.72]
Pre-treatment to 6FU	−0.35	0.64	0.57	[−1.60, 0.89]
Pre-treatment to 12 FU	−0.54	0.66	0.41	[−1.83, 0.74]
*Past-Session Recall x Treatment Condition*				
Pre-treatment to post-treatment	−1.38	0.78	0.08	[−2.91, 0.16]
Pre-treatment to 6FU	−0.63	0.78	0.42	[−2.15, 0.91]
Pre-treatment to 12 FU	−0.87	0.81	0.28	[0.71, 2.45]

Model 3: Cumulative Recall on WHODAS				
Random Effects	Variance	SD		
Patient	192.20	13.86		
Residual	109.20	10.45		
Fixed Effects	b	SE	p	95% CI
*Cumulative Recall*				
Pre-treatment to post-treatment	0.74	1.09	0.49	[−1.38, 2.86]
Pre-treatment to 6FU	−1.84	1.08	0.09	[−3.99, 0.28]
Pre-treatment to 12 FU	−3.09	1.12	**<0.01**	[−5.27, −0.91]
*Cumulative Recall x Treatment Condition*				
Pre-treatment to post-treatment	−2.30	1.31	0.08	[−4.85, 0.26]
Pre-treatment to 6FU	−0.34	1.30	0.79	[−2.88, 2.19]
Pre-treatment to 12 FU	3.11	1.33	**0.02**	[0.51, 5.70]

Model 4: Past-Session on WHODAS				
Random Effects	Variance	SD		
Patient	195.00	13.97		
Residual	109.60	10.47		
Fixed Effects	b	SE	p	95% CI
*Past-Session Recall*				
Pre-treatment to post-treatment	0.45	1.00	0.66	[−1.52, 2.42]
Pre-treatment to 6FU	−1.57	1.00	0.12	[−3.54, 0.39]
Pre-treatment to 12 FU	−2.68	1.05	**0.01**	[−4.72, −0.63]
*Past-Session Recall x Treatment Condition*				
Pre-treatment to post-treatment	−0.70	1.25	0.58	[−3.14, 1.74]
Pre-treatment to 6FU	0.90	1.24	0.47	[−1.52, 3.33]
Pre-treatment to 12 FU	2.26	1.28	0.08	[−0.24, 4.75]

***Note.*** IDS-SR = Inventory of Depressive Symptomatology, Self-Report; WHO-DAS = World Health Organization Disability Assessment Schedule.

## Data Availability

Data will be made available on request.
